# Inhibition of PTEN promotes osteointegration of titanium implants in type 2 diabetes by enhancing anti-inflammation and osteogenic capacity of adipose-derived stem cells

**DOI:** 10.3389/fbioe.2024.1358802

**Published:** 2024-02-15

**Authors:** Guanhua Zhang, Shuang Song, Zijun Chen, Xiangdong Liu, Jian Zheng, Yuxi Wang, Xutao Chen, Yingliang Song

**Affiliations:** ^1^ Department of Implant Dentistry, School of Stomatology, State Key Laboratory of Oral and Maxillofacial Reconstruction and Regeneration and National Clinical Research Center for Oral Diseases and Shaanxi Clinical Research Center for Oral Diseases, The Fourth Military Medical University, Xi’an, Shaanxi, China; ^2^ College of Stomatology, Key Laboratory of Shaanxi Province for Craniofacial Precision Medicine Research, College of Stomatology, Xi’an Jiaotong University, Xi’an, China; ^3^ Department of Immunology, Fourth Military Medical University, Xi’an, Shaanxi, China

**Keywords:** type 2 diabetes mellitus, *Pten*, MiR-140-3p, adipose-derived stem cells, osteogenesis

## Abstract

**Background:** The low osteogenic differentiation potential and attenuated anti-inflammatory effect of adipose-derived stem cells (ADSCs) from animals with type 2 diabetes mellitus (T2DM) limits osseointegration of the implant. However, the underlying mechanisms are not fully understood.

**Methods:** Western blotting and qRT-PCR analyses were performed to investigate the effects of PTEN on the osteogenic capacity of ADSCs of T2DM rats (TADSCs). We conducted animal experiments in T2DM-Sprague Dawley (SD) rats to evaluate the osteogenic capacity of modified TADSC sheets *in vivo*. New bone formation was assessed by micro-CT and histological analyses.

**Results:** In this study, adipose-derived stem cells of T2DM rats exhibited an impaired osteogenic capacity. RNA-seq analysis showed that PTEN mRNA expression was upregulated in TADSCs, which attenuated the osteogenic capacity of TADSCs by inhibiting the AKT/mTOR/HIF-1α signaling pathway. miR-140-3p, which inhibits PTEN, was suppressed in TADSCs. Overexpression or inhibition of PTEN could correspondingly reduce or enhance the osteogenic ability of TADSCs by regulating the AKT/mTOR/HIF-1α signaling pathway. TADSCs transfected with PTEN siRNA resulted in higher and lower expressions of genes encoded in M2 macrophages (Arg1) and M1 macrophages (iNOS), respectively. In the T2DM rat model, PTEN inhibition in TADSC sheets promoted macrophage polarization toward the M2 phenotype, attenuated inflammation, and enhanced osseointegration around implants.

**Conclusion:** Upregulation of PTEN, which was partially due to the inhibition of miR-140-3p, is important for the attenuated osteogenesis by TADSCs owing to the inhibition of the AKT/mTOR/HIF-1α signaling pathway. Inhibition of PTEN significantly improves the anti-inflammatory effect and osteogenic capacity of TADSCs, thus promoting peri-implant bone formation in T2DM rats. Our findings offer a potential therapeutic approach for modifying stem cells derived from patients with T2DM to enhance osseointegration.

## Graphical Abstract

**Figure:**
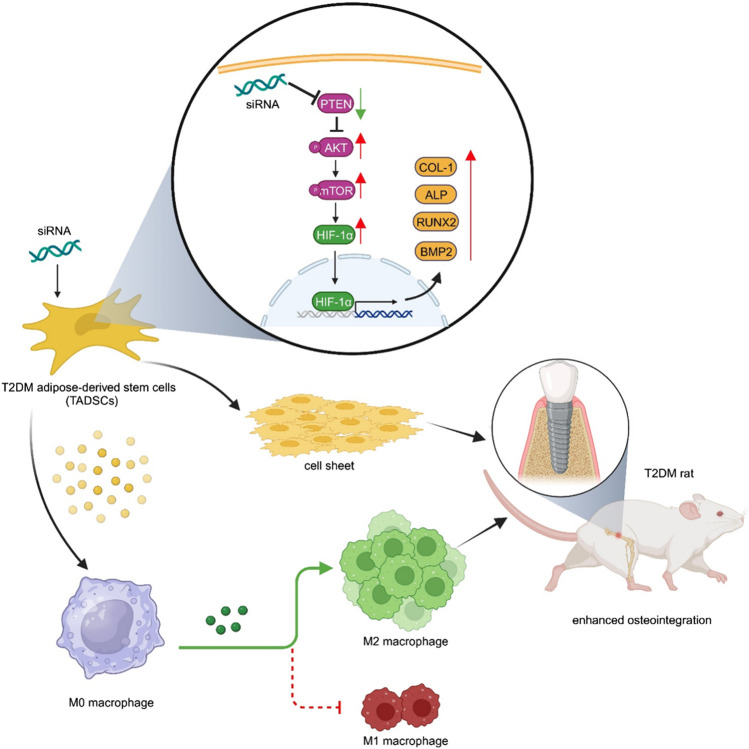
Inhibition of PTEN simultaneously improved the anti-inflammatory effect and osteogenic capacity of TADSCs, and ultimately promoted peri-implant bone formation in T2DM rats.

## 1 Introduction

For individuals with missing teeth, implant restorations are frequently performed owing to the consistency and reliability of this restorative procedure (Buser et al., 2017). Due to a chronic inflammatory microenvironment and poor bone metabolism (Rask-Madsen and King, 2013; Ko et al., 2015; Yun, 2015; Zhu et al., 2021), patients with type 2 diabetes mellitus (T2DM) have a substantially greater risk of implant failure than normal individuals (Niang et al., 2011; Shi et al., 2022). Therefore, it is necessary to find strategies to improve osseointegration as well as implant success in diabetic patients.

Adipose-derived stem cells (ADSCs), a type of mesenchymal stem cells, are promising for stem cell-based bone tissue engineering given their immune regulation capacity and multipotential differentiation capacity (Meliga et al., 2007; Li et al., 2020; Mutschall et al., 2020; Rochette et al., 2020; Wang L. et al., 2022; Bhattacharjee et al., 2022). The clinical application of ADSCs is constrained by an attenuated anti-inflammatory effect and osteogenic differentiation ability of ADSCs derived from patients with T2DM compared with normal individuals (Rawal et al., 2020). However, the mechanisms underlying are not well understood.

The phosphatidylinositol 3 kinase/protein kinase B (PI3K/AKT) signaling pathway is important in the development, inflammatory regulation, and bone formation in T2DM. In mouse models of obesity or T2DM, the PI3K/AKT signaling pathway is inhibited (Huang et al., 2018). AKT activation is essential for M2 polarization as the PI3K/AKT signaling pathway promotes the expression of several cytokines, including transforming growth factor-β (TGF-β), interleukin-10 (IL-10), and bone morphogenetic protein-7 (BMP-7), which contributes to the polarization of macrophage toward the M2 phenotype (Ruckerl et al., 2012; Byles et al., 2013; Covarrubias et al., 2015; Vergadi et al., 2017). M2 macrophages secrete osteogenic factors that stimulate the differentiation and activation of osteogenic-related cells and promote bone regeneration (Munoz et al., 2020). The PI3K/AKT signaling pathway is closely related to the osteogenic process by inhibiting the degradation of the osteogenic-related proteins, promoting the proliferation of osteoblast-associated cells, and increasing protein stability and transcriptional activity of Runt-related transcription factor 2 (RUNX2) (Choi et al., 2014; Manning and Toker, 2017; Price, 2006). Therefore, targeting the PI3K/AKT signaling pathway may promote osteogenesis.

Phosphatase and tensin homolog (PTEN) was first identified as a tumor suppressor with regulatory functions for growth and survival (Worby and Dixon, 2014). As the primary negative regulator of the PI3K/AKT signaling pathway, the role of PTEN as an immunomodulatory factor and an osteogenesis regulator has received considerable research traction in recent years (Vergadi et al., 2017; Lv et al., 2020; Xia et al., 2020). However, little is known about the immunomodulation and osteogenic function of PTEN in TADSCs and the PTEN expression difference in the expression of PTEN between normal ADSCs and ADSCs of T2DM rats (TADSCs).

In this study, we provide evidence that PTEN inhibition could promote M2 macrophage polarization and the osteogenic capacity of TADSCs. *In vivo*, TADSC sheets with low PTEN expression could simultaneously promote M2 polarization and peri-implant bone regeneration, ultimately enhancing peri-implant osseointegration in T2DM Sprague Dawley (SD) rats. Our findings provide a potent stem cell-based bone tissue engineering material to enhance peri-implant osteogenesis and propose a novel therapeutic approach that targets PTEN for enhancing implant osseointegration of patients with T2DM.

## 2 Material and methods

### 2.1 Animals and ethical considerations

All experiments were approved by the Ethics Committee of the School of Stomatology, the Fourth Military Medical University. All procedures during feeding and experimentation strictly followed the guidelines of the Institutional Animal Care and Use Committee of China. Twenty 8-week-old male SD rats (Chengdu Dossy Experimental Animals Co., Ltd., Chengdu, China) were acquired to isolate ADSCs for subsequent animal experiments. The standard pathogen-free settings of 25°C, 55% humidity, and 12 h of light followed by 12 h of darkness were maintained. A high-sucrose and high-fat diet was used to induce insulin resistance in 24 randomly chosen rats. Blood glucose and body weight were monitored after injection of streptozotocin (STZ, Sigma, St. Louis, MO) (30 mg/kg). Rats with blood glucose levels higher than 16.7 mmol/L were successfully constructed to establish the T2DM model and included in subsequent experiments. One rat which did not express diabetes and three rats dead after injection of STZ were excluded from the experiment.

### 2.2 Isolation, culture, and sheet fabrication of ADSCs

ADSCs or TADSCs were isolated surgically from the inguinal area of the subcutaneous adipose tissue of normal or T2DM rats and digested with an equal volume of 0.2% type I collagenase at 37°C for 60 min. Cells were filtered using a 200-mesh and centrifuged at 1000 g for 5 min. After removing the supernatant, the precipitate was resuspended in complete alpha-modified eagle medium (MEM, 10% fetal bovine serum (FBS) from Gibco, USA, and 1% penicillin/streptomycin was obtained from Hyclone, USA) and cultured in T75 culture flasks in an incubator (37°C, 5% CO_2_). The culture medium was changed every 48 h. Cells were passaged after reaching 80% confluence. The cells used in the following experiments were collected from the third passage. Cells from the third passage of TADSCs were seeded in six-well cell culture plates at a density of 1 × 10^6^ cells/well. After reaching approximately 100% confluence, the basal medium was replaced with α-MEM (Gibco, Thermo Fisher, Waltham, MA, USA) containing 10% FBS (Gibco, Thermo Fisher, Waltham, MA, United States), cell sheet-inducing medium containing 1% penicillin/streptomycin (HyClone, Cytiva, Marlborough, MA, United States), and 50 mg/mL vitamin C (Sigma-Aldrich, St. Louis, MO, United States). TADSCs were grown for 7–10 days, and the medium was changed every 2–3 days. When the curled edge of the cell membrane appeared in the culture plate, the entire cell sheet was gently peeled off using a pair of tweezers. The TADSC sheet was moist during stripping.

### 2.3 Immunocharacterization of ADSCs and TADSCs

Approximately 1 × 10^6^ ADSCs from the third passage were fixed in 4% paraformaldehyde (PFA) for 15 min. ADSCs and TADSCs were incubated with phycoerythrin (PE)- or fluorescein isothiocyanate (FITC)-conjugated monoclonal antibodies against CD29 (BioLegend, San Diego, CA, United States), CD44 (BioLegend, San Diego, CA, United States), CD45 (BioLegend, San Diego, CA, United States), and CD105 (BioLegend, San Diego, CA, United States) at room temperature (RT) in the dark for 1 h. Subsequently, both ADSCs and TADSCs were examined by flow cytometry (DxFLEX, Beckman Coulter, Brea, CA, United States). Monoclonal antibodies, including CD29-PE, CD44-PE, and CD105-PE, were used to identify the mesenchymal lineage. Simultaneously, hematopoietic and angiogenic phenotypes were excluded using CD45-FITC antibodies.

### 2.4 Examination of ADSC osteogenic differentiation potential

Cells were seeded in six-well culture plates to examine the osteogenic differentiation potential of ADSCs. When the cells reached 80% confluence, the medium was replaced with an osteogenic induction medium, followed by induction with fresh medium every 3 days. Osteogenic induction media were obtained from Cyagen Biosciences (RAXMD-90021). Alkaline phosphatase (ALP) staining (Beyotime, Shanghai, China) was performed after 7 days and 1% Alizarin Red S (ARS) solution (Solarbio, Beijing, China) was used to assess calcium deposition 21 days after osteogenic induction.

### 2.5 PTEN siRNA and cDNA transfection

Transfection with siRNA was performed for *Pten* gene silencing using siRNA synthesized by GenePharma, with the following sequence: GCC​AGC​UAA​AGG​UGA​AGA​UTT (si-*Pten*). TADSCs were grown on 6-well plates in complete alpha-MEM until they reached 60%–70% confluence. Lipofectamine 3000 (Invitrogen, Waltham, MA) was used following the manufacturer’s protocol to transfect the blank, non-targeting siRNA as the negative control (si-NC) or *Pten* siRNA (si-*Pten*) in cells. Plasmids containing PTEN cDNA (over-*Pten*), empty plasmid (over-NC), or blank were transfected separately into TADSCs using Lipofectamine 3000 (Invitrogen, Waltham, MA) following the manufacturer’s instructions. Total RNA and protein were isolated at 24 and 48 h after transfection for qRT-PCR or western blotting analysis, respectively.

### 2.6 RNA isolation, cDNA synthesis, and quantitative real-time PCR

Total RNA was isolated using the TRIzol reagent (Invitrogen, Waltham, MA, United States), following the manufacturer’s instructions. Following optical density-based quantification, 1 µg of total RNA was synthesized into cDNA using the PrimeScript™ RT Reagent Kit (Takara, Kyoto, Japan). Quantitative real-time polymerase chain reaction (qRT-PCR) analysis was performed using the SYBR Premix Ex Taq™ II Kit (Takara) on ABI 7500, Thermo Fisher system using the following conditions: denaturation for 3 min at 95°C, 40 rounds annealing for 10 s at 95°C, and extension for 30 s at 60°C. The internal controls for mRNAs and miR-140-3p were GAPDH and U6, respectively. qRT-PCR was performed using primer sequences synthesized by Sangon Biotech Shanghai Co., Ltd., Shanghai, China (Table 1).

**TABLE 1:** Primer sequences used in qRT-PCR to detect ADSC genes.

ADSC genes	Forward primer 5′→3′	Reverse primer 5ʹ→3′
*Hif-1α*	CCG​CCA​CCA​CCA​CTG​ATG​AAT​C	GTG​AGT​ACC​ACT​GTA​TGC​TGA​TGC​C
*Alp*	CAA​CGA​GGT​CAT​CTC​CGT​GAT​G	TAC​CAG​TTG​CGG​TTC​ACC​GTG​T
*Runx2*	CCC​AGT​ATG​AGA​GTA​GGT​GTC​C	GGG​TAA​GAC​TGG​TCA​TAG​GAC​C
*Col-1*	TGT​TGG​TCC​TGC​TGG​CAA​GAA​TG	GTC​ACC​TTG​TTC​GCC​TGT​CTC​AC
*Gapdh*	CAA​GTT​CAA​CGG​CAC​AGT​CA	CCA​TTT​GAT​GTT​AGC​GGG​AT
*Bmp2*	GAG​GAG​AAG​CCA​GGT​GTC​T	GTC​CAC​ATA​CAA​AGG​GTG​C
*mTOR*	CCT​TCG​TGC​CTG​TCT​GAT​TCT​TAC​C	GCT​GCT​GCT​GGG​TGA​TTT​CCT​C
*Pten*	GGG​AAA​GGA​CGG​ACT​GGT​GTA​ATG	CAC​AGG​CAA​TGG​CTG​AGG​GAA​C
*Akt*	AGG​CAT​CCC​TTC​CTT​ACA​GC	CAG​CCC​GAA​GTC​CGT​TAT​CT
*Arg1*	AAG​AAA​AGG​CCG​ATT​CAC​CT	CAC​CTC​CTC​TGC​TGT​CTT​CC
*iNOS*	CTA​CCT​ACC​TGG​GGA​ACA​CCT​GGG	GGA​GGA​GCT​GAT​GGA​GTA​GTA GCGG
*IL-10*	AGC​CTT​ATC​GGA​AAT​GAT​CCA​GT	GGC​CTT​GTA​GAC​ACC​TTG​GT
*IL-1β*	TGT​GGC​AGC​TAC​CTA​TGT​CT	GGG​AAC​ATC​ACA​CAC​TAG​CA
*TNF-α*	GGC​GTG​TTC​ATC​CGT​TCT​CT	CCC​AGA​GCC​ACA​ATT​CCC​TT
*CD206*	TCA​ACT​CTT​GGA​CTC​ACG​GC	ATG​ATC​TGC​GAC​TCC​GAC​AC
miR-140-3p	GCG​GCT​ACC​ACA​GGG​TAG​AA	ACT​GCA​GGG​TCC​GAG​GTA​TT
U6	AGC​ACA​TAT​ACT​AAA​ATT​GGA​ACG​AT	ACT​GCA​GGG​TCC​GAG​GTA​TT

### 2.7 RNA-sequencing analysis

The total RNA was isolated using TRIzol assay. Then RNA-sequencing assay was performed by 2100 RNA Nano 6000 Assay Kit (Agilent Technologies, CA, United States) after that the qualification of RNA met the requirement. Top 10 most significantly different genes were displayed by heat map. Then, GO (Gene Ontology) and KEGG (Kyoto Encyclopedia of Genes and Genomes) analyses were performed for the differently expressed genes between ADSCs and TADSCs.

### 2.8 Protein extraction and western blotting

Protein isolation and quantification were performed using the bicinchoninic acid (BCA) protein assay (Beyotime). Total protein extraction from the bone tissue was performed following the manufacturer’s instructions (Invent Minute™, Eden Prairie, United States). Protein samples in equal quantities (25 μg) were separated on a 10% sodium dodecyl sulfate-polyacrylamide gel and transferred onto a polyvinylidene difluoride membrane (Millipore, Beijing, China). Subsequently, the membrane was blocked for 1 h in fat-free milk (5%) in TBS-T (50 mmol/L Tris, pH 7.5; 150 mmol/L NaCl; 0.05% Tween-20). Subsequently, the membrane was incubated with the following primary antibodies at 4°C: PTEN (1:2000, ab267787, Cambridge, UK), HIF-1α (1:2000, ab179483; Abcam, Cambridge, UK), p-AKT (1:2000; Cell Signaling Technology, Danvers, MA, United States; #4060), total-AKT (1:2000; Cell Signaling Technology; #4685), p-mTOR (1:2000, ab109268; Abcam), total-mTOR (1:2000, ab32028; Abcam), RUNX2 (ab236639, 1:2000; Abcam), COL-1 (1:2000, ab260043; Abcam), ALP (1:2000, #C48608, SAB), BMP2 (1:2000, ab284387, abcam), iNOS (1:2000, ab178945, abcam), ARG1 (1:2000, ab203490, abcam) or GAPDH (1:10,000, ab181602; Abcam) diluted in TBS-T. The membrane was washed thoroughly thrice in TBS-T (5 min/wash) at RT and incubated for 1 h with a secondary antibody (#L3012, SAB, Maryland 20770, United States), followed by washing in TBS-T. Finally, protein bands were visualized using a chemical discharge imaging system (Bio-Rad GelDoc XR+, Hercules, CA, United States). The ImageJ software (version 1.8.0.112, National Institutes of Health, New York, United States) was utilized to determine the gray value of the target protein and the internal reference protein (GAPDH). The expression of the target protein was normalized against the level of GAPDH.

### 2.9 Co-culture of TADSCs and macrophages

TADSCs from the third passage were transfected with blank, non-targeting siRNA, empty plasmid, *Pten* siRNA, and *Pten* cDNA plasmid. After 24 h, the five groups of TADSCs were seeded in the upper chamber at a density of 1.5 × 10^4^ cells/well. M0 macrophages were seeded at the bottom of the chamber. After 48 h of co-culture, protein and RNA were extracted from the macrophages or they were used for immunofluorescence assay.

### 2.10 Surgical procedure and treatment

All animal experiments were approved by the Ethics Committee of the School of Stomatology, the Fourth Military Medical University (kq-024). The experiment comprised five groups (*n* = 5 in each group): 1) blank group: T2DM rats with implantation only, 2) si-NC group: T2DM rats implanted with TADSC sheets transfected with the non-targeting siRNA construct, 3) over-NC group: T2DM rats implanted with TADSC sheets transfected with the empty plasmid, 4) si-*Pten* group: T2DM rats implanted with TADSC sheets transfected with siRNA of *Pten*, 5) over-*Pten* group, T2DM rats implanted with TADSC sheets transfected with cDNA of *Pten*. The implants used in this study were from Kontour Medica (Xi’an, China) with the following dimensions: length, 5 mm and diameter, 2 mm. Rats were anesthetized with 1% sodium pentobarbital solution (45 mg/kg rat weight). During the operation, the animals were placed on heating pads to avoid hypothermia. A longitudinal incision of 1 cm was made on the lateral side of the knee joint after shaving both hind limbs. The blade cut through the skin and muscularis and directly reached the bone surface, consequently pulling the ligament from the outside to the inside, resulting in the exposure of the femoral condyle. A fissure bur and a round bur were used for preparing the implant cavity on the femoral epiphysis. The TADSC sheet was wrapped around the implant and screwed into the cavity. Incisions were sutured carefully in layers. On the fourth and 14th days before sacrifice, alizarin red S (30 mg/kg) and calcein (20 mg/kg) (Sigma-Aldrich, St, Louis, MO) were sequentially administrated intraperitoneally. Animals were sacrificed 4 weeks after implantation, and the femurs were harvested for radiographic and histological analyses.

### 2.11 Micro-CT analysis

The specimens were fixed overnight in 4% PFA (*n* = 3 in each group) and subjected to microcomputed tomography (Siemens Inveon, Erlangen, Germany) analysis to evaluate osteointegration around the implant. The region of interest (ROI), approximately 200 μm away from the implant surface, was reconstructed into three-dimensional structures to analyze the related morphometric parameters of bone around implants, including the bone volume percentage (BV/TV), trabecular thickness (Tb.Th), trabecular number (Tb. N), and trabecular separation (Tb. Sp).

### 2.12 Sequential fluorescence labeling, VanGieson staining, and toluidine blue staining

The samples were subjected to dehydration using an ethanol gradient (75%–100%), methyl methacrylate infiltration, and resin embedding in poly-methyl-methacrylate. Each specimen was divided into sections, 300 μm in thickness, by cutting through the implant’s center, parallel to its long axis. Slices were subsequently ground to a thickness of 80 μm for imaging with a confocal laser scanning microscope (OLYMPUS, Tokyo, Japan). The mineral apposition rate (MAR) was determined to quantify the speed of osteoid mineralization among the five groupsduring the observation period. The sections were stained with toluidine blue and Van Gieson’s stain (VG staining). Bone-to-implant contact (BIC) and the bone area (BA) were evaluated using the ImageJ software (National Institutes of Health, Bethesda, MD). BIC was calculated as the ratio of the length of the implant’s surface to the contact interface between the bone and the implant. BA refers to the percentage of bone tissue between the adjacent threads of the implant.

### 2.13 H&E staining, Masson’s trichrome staining, and safranin O-fast green staining

Briefly, 10% EDTA solution (pH = 7.2) was used to decalcify the sample for 30 days at 37°C after fixing (Servicebio, Wuhan, China); the decalcification solution was replaced every 3 days. The samples were embedded in paraffin wax after decalcification, and the implant cavities were divided into continuous sections with a thickness of 3 μm each. Sections were stained with H&E or Masson’s trichrome stain to evaluate the development of osteogenesis. For Safranin O-fast green staining, the specimens were promptly washed with a mild acid solution for 10–15 s after immersing in solid green staining solution for 5 min. The specimens were stained with Safranin O-fast green (Solarbio, Beijing, China) for 5 min. Subsequently, 95% and 100% ethanol were used to dehydrate the specimens followed by sealing them with optical resin after treatment with xylene.

### 2.14 Immunofluorescence analysis

For immunofluorescence analysis, cells or 6 μm tissue sections were incubated for 14 h at 4°C with primary antibodies against iNOS (1:100, 18985-1-AP, Proteintech), ARG1 (1:100, 66129-1-Ig, Proteintech), followed by incubation with the corresponding Goat Anti-Mouse IgG (H+L) FITC-conjugated secondary antibodies (1:500, S0007, Affinity Biosciences, United States) or CoraLite594-conjugated Goat Anti-Rabbit IgG (H+L) (1:500, SA00013-4, Proteintech, China) at RT for 2 h. Nuclei were stained with DAPI. Immunofluorescence-stained sections and cells were examined and imaged using a laser scanning confocal microscope (NikonA1+/A1R+, Tokyo, Japan).

### 2.15 Statistical analysis

Quantitative data are described using the mean ± SD of at least three independent experiments, and statistical analyses were performed using GraphPad Prism 5.0 (GraphPad Software, San Diego, CA). Quantitative data of multiple groups were analyzed using one-way analysis of variance (ANOVA), and differences between two groups were analyzed using the *t-test*. A *p*-value less than 0.05 was considered statistically significant.

## 3 Results

### 3.1 Osteogenic capacity of TADSCs was impaired

**FIGURE 1:**
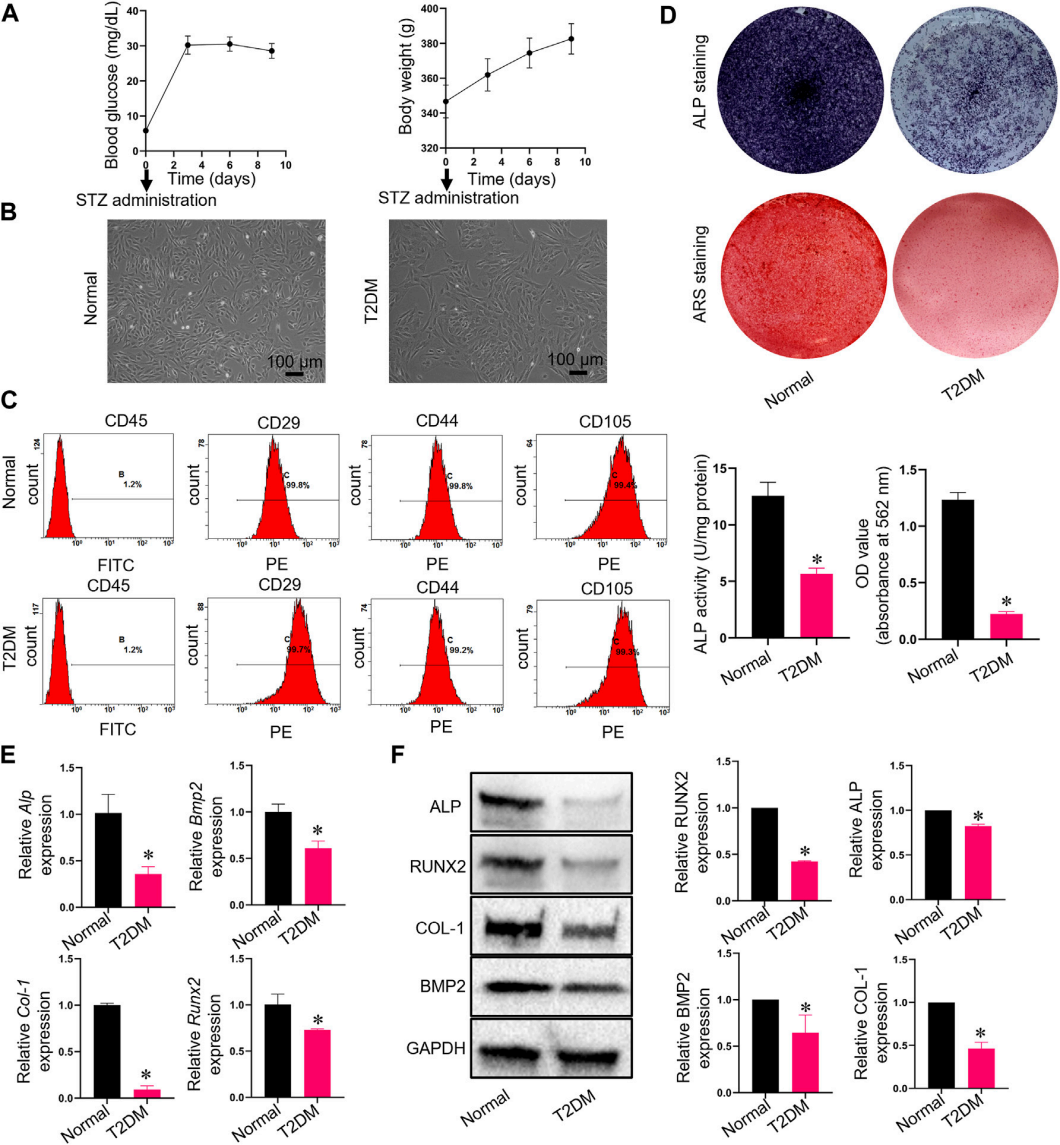
Characterization and the detection of osteogenic differentiation capacities of ADSCs and TADSCs. **(A)** Blood glucose and body weight after injection of STZ. **(B)** Representative image of ADSCs and TADSCs in passage 3. **(C)** Flow cytometry analysis for detrmining the immunophenotypic characteristics of ADSCs and TADSCs. **(D)** ALP and ARS staining after osteogenic induction for 7- or 21-days. **(E)** Expression of osteogenic-related genes in ADSCs and TADSCs. **(F)** Expression of osteogenesis-related proteins in ADSCs and TADSCs. All data are presented as the mean ± SD of at least three independent experiments; *p < 0.05.

We induced T2DM SD rats with STZ successfully and monitored blood glucose and body weight after injection of STZ (Figure 1A). We obtained and used cells from the third passage for experiments after isolating ADSCs or TADSCs from normal or male SD rats with T2DM. Both cell types showed a fibroblast-like spindle-shaped morphology (Figure 1B). Flow cytometry analysis showed that CD45 was not expressed in both groups, while staining for CD29, CD44, and CD105 were all positive (Figure 1C). At day 7, ALP activity—a feature of early-stage osteogenic differentiation of ADSCs—was determined. Compared to the T2DM group, ALP levels in the normal group were remarkably higher. Calcified nodules were subjected to ARS staining after 21 days of osteogenic differentiation. ADSCs showed greater mineral deposition compared to TADSCs. (Figure 1D). We extracted RNA and protein from both cell types to verify the observed phenomenon of diminished osteogenic capacity of TADSCs compared to ADSCs. Osteogenic-related genes, including *Alp*, *Runx2*, *Bmp2*, and *Col-1*, were all reduced in TADSCs (Figure 1E). Similarly, results of western blotting showed that the expression of osteogenic-related proteins, including ALP, RUNX2, bone morphogenetic protein-2 (BMP2), and type 1 collagen (COL-1) in TADSCs were all significantly downregulated compared to ADSCs (Figure 1F).

### 3.2 *Pten* expression was elevated in TADSCs

**FIGURE 2:**
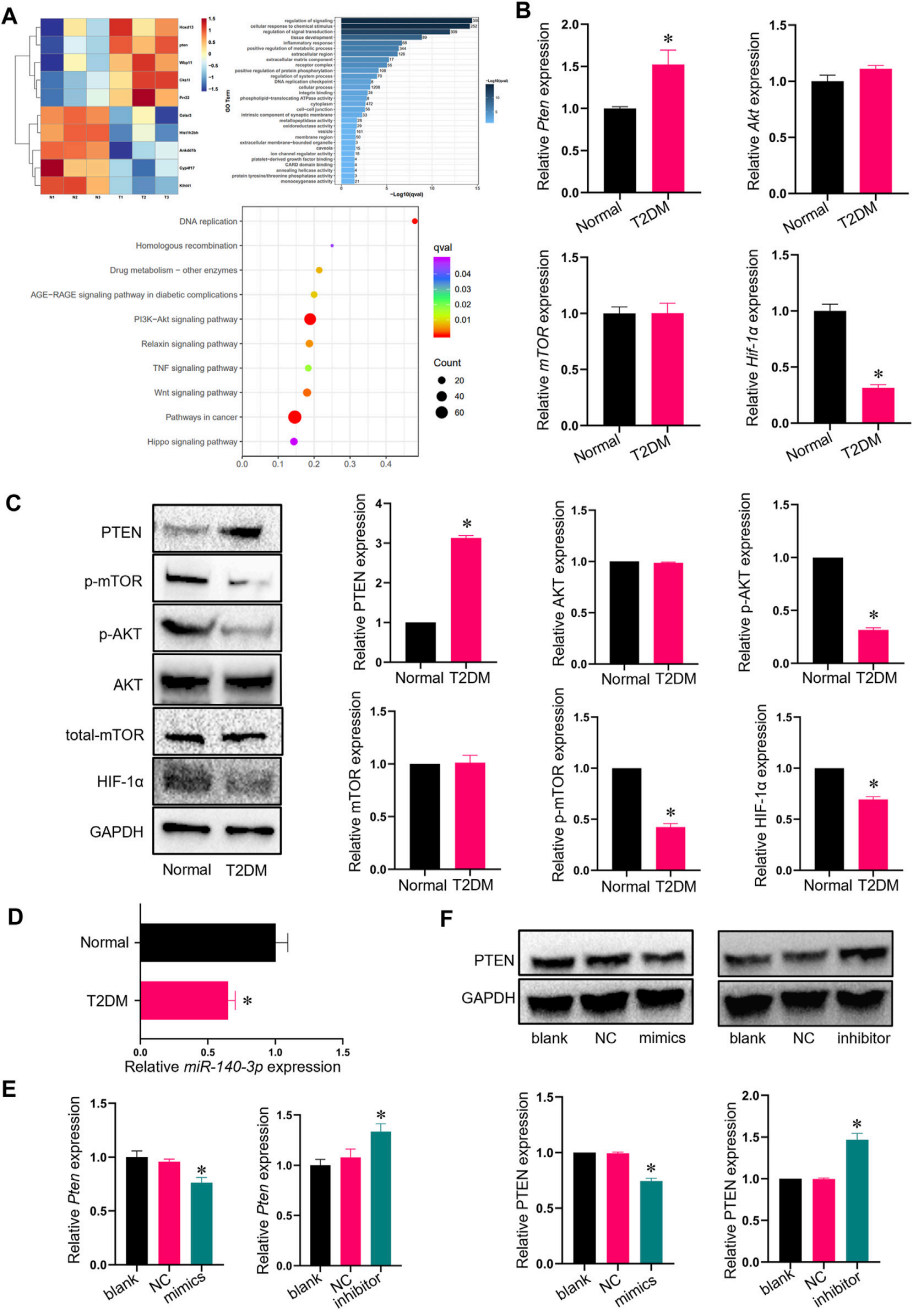
*Pten* expression is elevated in TADSCs. **(A)** Heatmap showing GO and KEGG analyses of RNA sequencing results. **(B)** mRNA expression of the genes involved in AKT signaling in ADSCs and TADSCs. **(C)** Protein expression of genes involved in AKT signaling in ADSCs and TADSCs. **(D)** Expression of miR-140-3p in ADSCs and TADSCs. **(E)** miRNA-140-3p expression in TADSCs after treatment with miR-140-3p mimics or inhibitors. **(F)** PTEN expression in TADSCs after treatment with miR-140-3p mimics or inhibitors. All data are presented as the mean ± SD of at least three independent experiments; *p < 0.05.

RNA-seq analysis was performed to identify differences in gene expression between ADSCs and TADSCs, which shed light on the molecular mechanisms underlying the impairment of osteogenic differentiation of TADSCs. A total of 46,528,282 and 46,650,918 clean reads were available after cleaning and quality control. *Pten* mRNA was considerably upregulated in TADSCs among significantly and differentially expressed genes between ADSCs and TADSCs (|fold change|>1 and *p* < 0.05) (Figure 2A). GO (Figure 2B) and KEGG pathway enrichment (Figure 2C) analyses were performed. In KEGG analysis, genes were significantly enriched in the PI3K/AKT signaling pathway. Considering that PTEN is a primary negative regulator and is located upstream of the PI3K/AKT signaling pathway, PTEN might majorly contribute to the impaired osteogenic capacity of TADSCs by modulating the PI3K/AKT signaling pathway. We conducted qRT-PCR to verify the high expression of *Pten* in TADSCs. *Pten* expression was significantly elevated in TADSCs compared to ADSCs. The AKT/mTOR/HIF-1α signaling pathway is important in promoting osteogenesis, with PTEN being a predominant negative regulator. We examined the mRNA expression of AKT, mTOR, and HIF-1α and the protein expression of total-AKT, p-AKT, total-mTOR, p-mTOR, and HIF-1α to investigate whether the AKT/mTOR/HIF-1α signaling pathway contributes to the reduced osteogenic capacity of TADSCs. The AKT/mTOR/HIF-1α signaling pathway was significantly inhibited in TADSCs compared to ADSCs (Figures 2D, E). We verified the expression of miR-140-3p, which directly inhibited PTEN, to determine the mechanism underlying high expression of PTEN in TADSCs. PTEN upregulation may be partially due to the inhibition of miR-140-3p in TADSCs (Figure 2F). We transfected TADSCs with miR-140-3p mimics and miR-140-3p inhibitors, respectively, to confirm the hypothesis and detect mRNA and protein expressions of miR-140-3p and PTEN. The mRNA level of miRNA-140-3p was significantly upregulated after overexpression of miRNA-140-3p, while the protein expression of PTEN was significantly downregulated. On the contrary, mRNA and protein expressions of PTEN were significantly upregulated after the transfection of the miRNA-140-3p inhibitor (Figures 2G, H). The upregulation of PTEN in TADSCs was at least partially due to the suppression of miR-140-3p.

### 3.3 Knockdown of *Pten* enhanced osteogenic capacity of TADSCs via AKT/mTOR/HIF-1α signaling pathway

**FIGURE 3:**
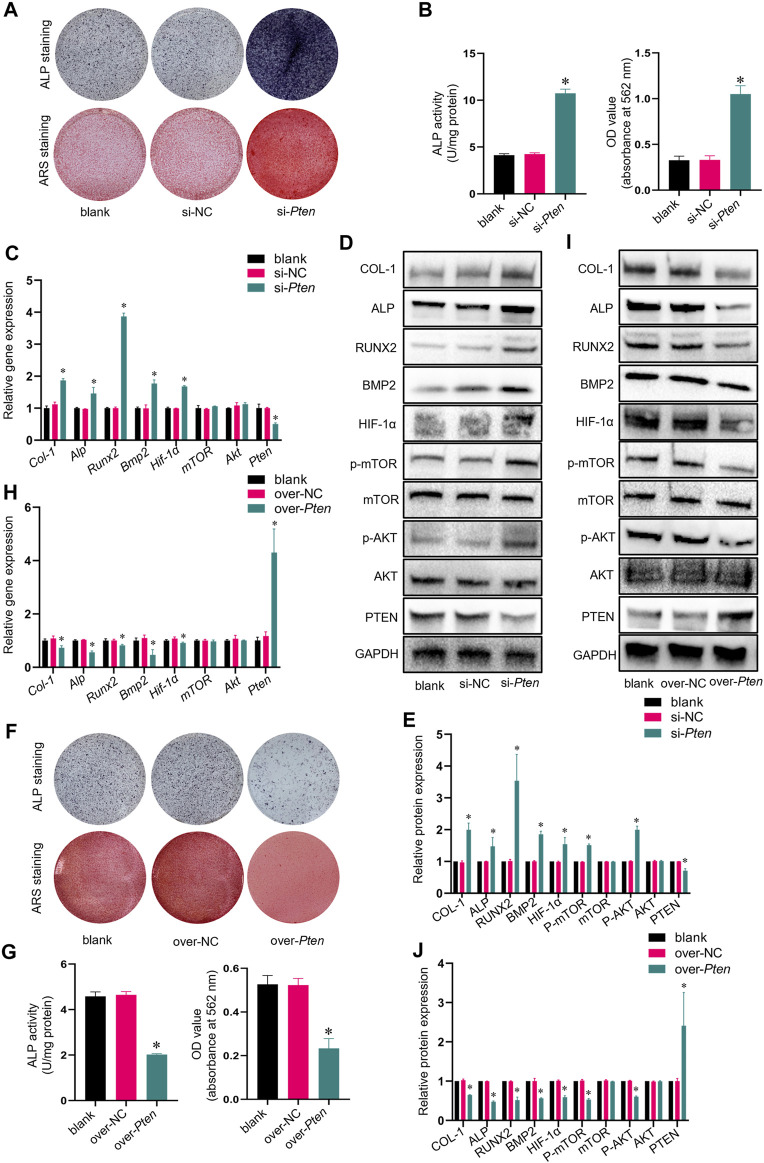
Knockdown of *Pten* enhances the osteogenic capacity of TADSCs through the AKT/mTOR/HIF-1α signaling pathway. **(A)** ALP and ARS staining after treatment with blank, nonsense *Pten* siRNA, and *Pten* siRNA after 7- or 21-day osteogenic induction. **(B)** Quantification of ALP and ARS staining results. **(C)** mRNA expression of osteogenic- and AKT/mTOR/HIF-1α signaling pathway-related genes in TADSCs after indicated treatments. **(D)** Expression of osteogenesis- and AKT/mTOR/HIF-1α signaling pathway-related proteins in TADSCs after different treatments. **(E)** Quantification of western blotting results. **(F)** ALP and ARS staining after treatment with blank, empty cDNA plasmid and *Pten* cDNA after 7- or 21-day osteogenic induction. **(G)** Quantification of ALP and ARS staining results. **(H)** Expression of osteogenesis- and AKT/mTOR/HIF-1α signaling pathway-related proteins in TADSCs after different treatments. **(I)** Expression of osteogenesis- and AKT/mTOR/HIF-1α signaling pathway-related proteins in TADSCs after different treatments. **(J)** Quantification of western blotting results. All data are presented as the mean ± SD of at least three independent experiments; *p < 0.05.

siRNA was utilized to inhibit the *Pten* mRNA expression in TADSCs to assess the effect of PTEN on osteogenic differentiation capacity in TADSCs. After 7- or 21-day induction, the osteogenic capacity of TADSCs in the si-*Pten* group was enhanced (Figures 3A, B). The mRNA level of *Pten* decreased significantly after transfection; in contrast, the mRNA levels of *Hif-1α* and osteogenic genes, including *Alp*, *Runx2*, *Bmp2*, and *Col-1* were significantly upregulated, while those of *mTOR* and *Akt* were not significantly different compared to the blank and negative control. Similarly, the protein expressions of HIF-1α, p-AKT, p-mTOR, ALP, RUNX2, BMP2, and COL-1 in the si-PTEN group were upregulated, while those of total-mTOR and total-AKT were not significantly changed (Figures 3C–E). Plasmids containing PTEN cDNA, or empty plasmid were transfected in TADSCs for further validation. According to the results of ARS and ALP staining, the osteogenic capacity of TADSCs was attenuated (Figures 3F, G). Western blotting showed elevated *Pten* protein expression in the over-*Pten* group. In contrast, the mRNA expressions of *Hif-1α*, *Alp*, *Runx2*, *Bmp2*, and *Col-1* were significantly downregulated. Similarly, the protein expressions of HIF-1α, p-AKT, p-mTOR, ALP, RUNX2, BMP2, and COL-1 were suppressed (Figures 3H, I). Taken together, silencing PTEN could remarkably activate the AKT/mTOR/HIF-1α signaling pathway and enhance the osteogenic capacity of TADSCs.

### 3.4 Co-culturing macrophage with TADSCs of *Pten* low expression further promoted M2 macrophage polarization *in vitro*

**FIGURE 4:**
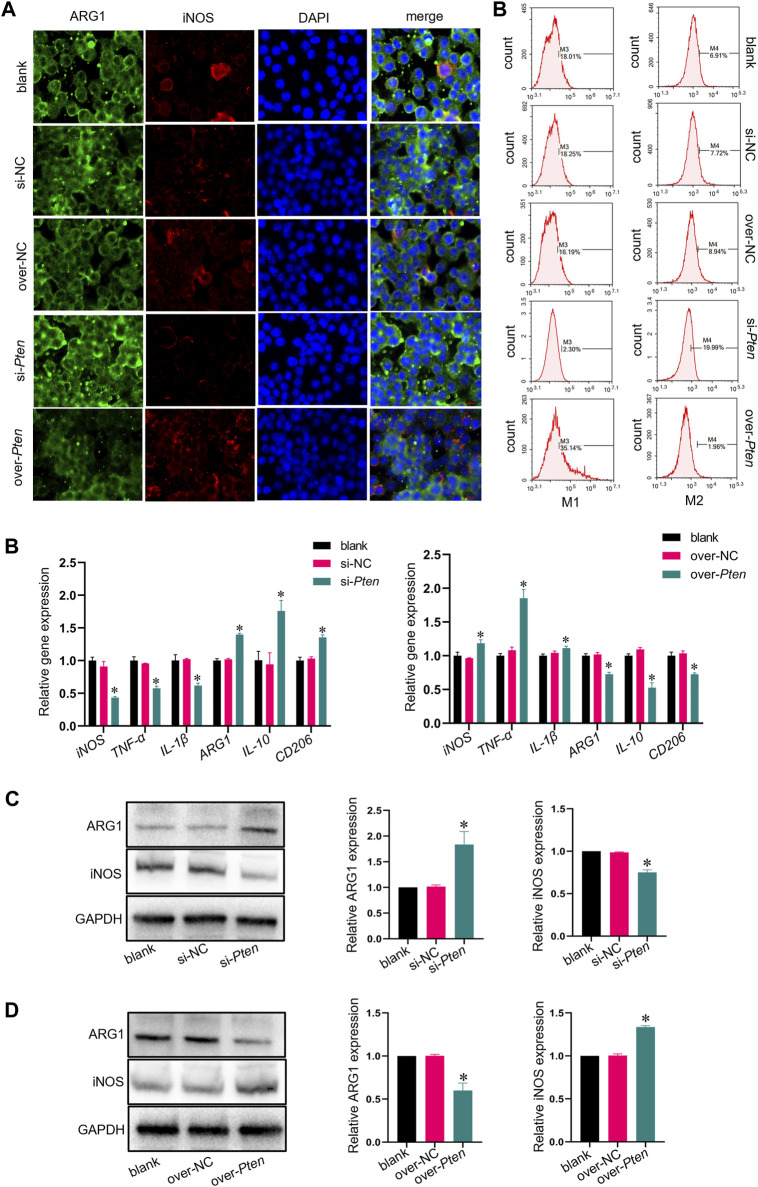
Co-culture of macrophage and TADSCs with low *Pten* expression promotes M2 macrophage polarization *in vitro*. **(A)** Representative images of macrophages by immunofluorescence staining (ARG1 in green, iNOS in red, and nuclei in blue; scale bar = 20 μm). **(B)** Marker genes of M1 and M2 macrophages after co-culture with differently treated TADSCs. **(C)** Protein levels of M1 and M2 macrophage markers after co-culture with differently treated TADSCs. **(D)** Quantification of western blotting results. All data are presented as the mean ± SD of at least three independent experiments; *p < 0.05.

TADSCs treated with *Pten* siRNA and M0 macrophages were co-cultured to explore whether TADSCs modified by *Pten* siRNA could promote the M2 polarization of macrophages *in vitro*. The si-*Pten* group showed an obviously decreased ratio of cells with inducible nitric oxide synthase (iNOS) (M1 macrophage marker) and an increased ratio of cells with Arginase-1 (ARG1) (M2 macrophage marker). The over-*Pten* group showed the lowest ratio of M2 cells and the highest ratio of M1 cells (Figure 4A). qRT-PCR was conducted for further demonstration of the effect of TADSCs modified by P*ten* siRNA on promoting M2 polarization of macrophages *in vitro*. *Pten* inhibition in TADSCs resulted in the highest expression of genes encoding M2 macrophages, including *Arg1*, *CD206*, and *IL-10*, and the lowest of those encoding M1 macrophages, such as *iNOS*, *TNF-α*, and *IL-1β*. On the contrary, TADSCs showing *Pten* overexpression exhibited lower expression of genes encoding M2 macrophages and higher of those encoding M1 macrophages (Figure 4B). Western blotting showed that ARG1 expression in the si-*Pten* group was significantly higher compared to the other groups (Figures 4C, D). In summary, the ability of TADSCs treated with *Pten* siRNA to promote the M2 polarization of macrophages was significantly enhanced *in vitro*.

### 3.5 TADSC sheets of *Pten* low expression enhanced osteointegration around titanium implants in T2DM rats

**FIGURE 5:**
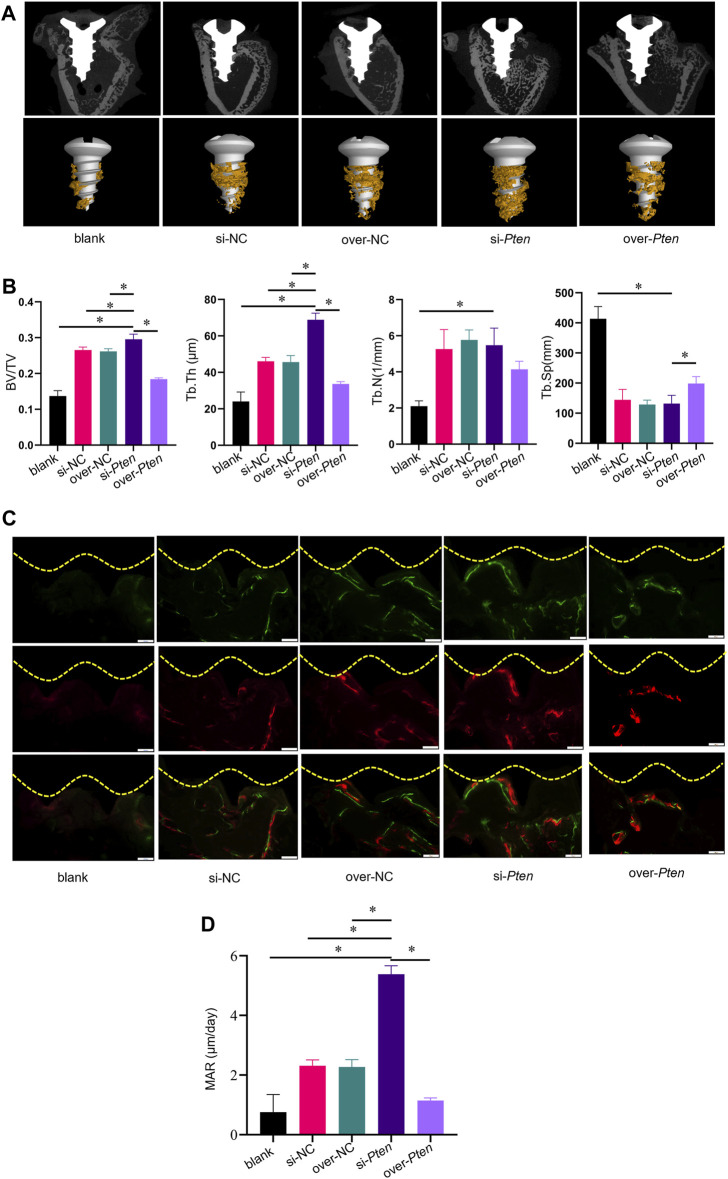
TADSC sheets with low *Pten* expression enhance osteointegration around titanium implants in T2DM rats. **(A)** Micro-CT analysis of two-dimensional scanned images of rat femoral condyles and reconstructed three-dimensional structures of the region of interest (ROI), including the bone-to-implant contact area (yellow). **(B)** Histomorphometric parameters analysis including BV/TV, Tb. Th, Tb. N, and Tb. Sp. **(C)** New bone formation is indicated by double fluorescence labeling with Alizarin red stain (red) and Calcein (green) in rat femoral condyles. **(D)** Quantitative analysis of the rate of osteoid mineralization by MAR. *p < 0.05.

Titanium implants (length = 5 mm, diameter = 2 mm) wrapped with TADSC sheets transfected with *Pten* siRNA were inserted into the femoral condyles of SD rats with T2DM to evaluate the role of PTEN in regulating the osteogenic capacity of TADSCs *in vivo*. Five experimental groups were set, including blank (without TADSC sheets), si-NC (TADSC sheets treated with the negative control of *Pten* siRNA); over-NC (TADSC sheets treated with the negative control of *Pten* overexpressing plasmids); si-*Pten* (TADSC sheets treated with *Pten* siRNA); over-*Pten* (TADSC sheets treated with *Pten* overexpression plasmids). Micro-computed tomography (Micro-CT) was performed, and scans were reconstructed to analyze the structure of regenerative bone around implants with an ROI 200 μm away from the implant surface. The osteointegration area around implants is shown in yellow. The analysis of bone microstructure in the ROI showed significantly the highest BV/TV and Tb. Th in the si-*Pten* group. The over-*Pten* group showed lower BV/TV and Tb. Th compared to the si-*Pten* group and both negative control groups but the value was higher than that of the blank group. However, the blank group without TADSC sheets had the minimum bone-to-implant contact area (Figures 5A, B). Sequential fluorescent labeling was performed to investigate whether TADSC sheets treated with *Pten* siRNA could accelerate osteoid mineralization after implantation. Alizarin red (red) and Calcein (green) were intraperitoneally injected sequentially at four and 14 days before sacrifice, respectively. The si*-Pten* group had the largest area of fluorescent labeling lines around implants, indicative of the highest regenerative bone formation among the five groups. Knocking down *Pten* in TADSC sheets considerably promoted osteointegration in T2DM rats. The si-*Pten* group showed the fastest osteoid mineralization rate, which was evident from the MAR. However, as expected, *Pten* overexpression in TADSC sheets greatly reduced the rate of mineral apposition, and consequently, new bone formation (Figures 5C, D).

### 3.6 Histological analyses of osseointegration and new bone formation around implants

**FIGURE 6:**
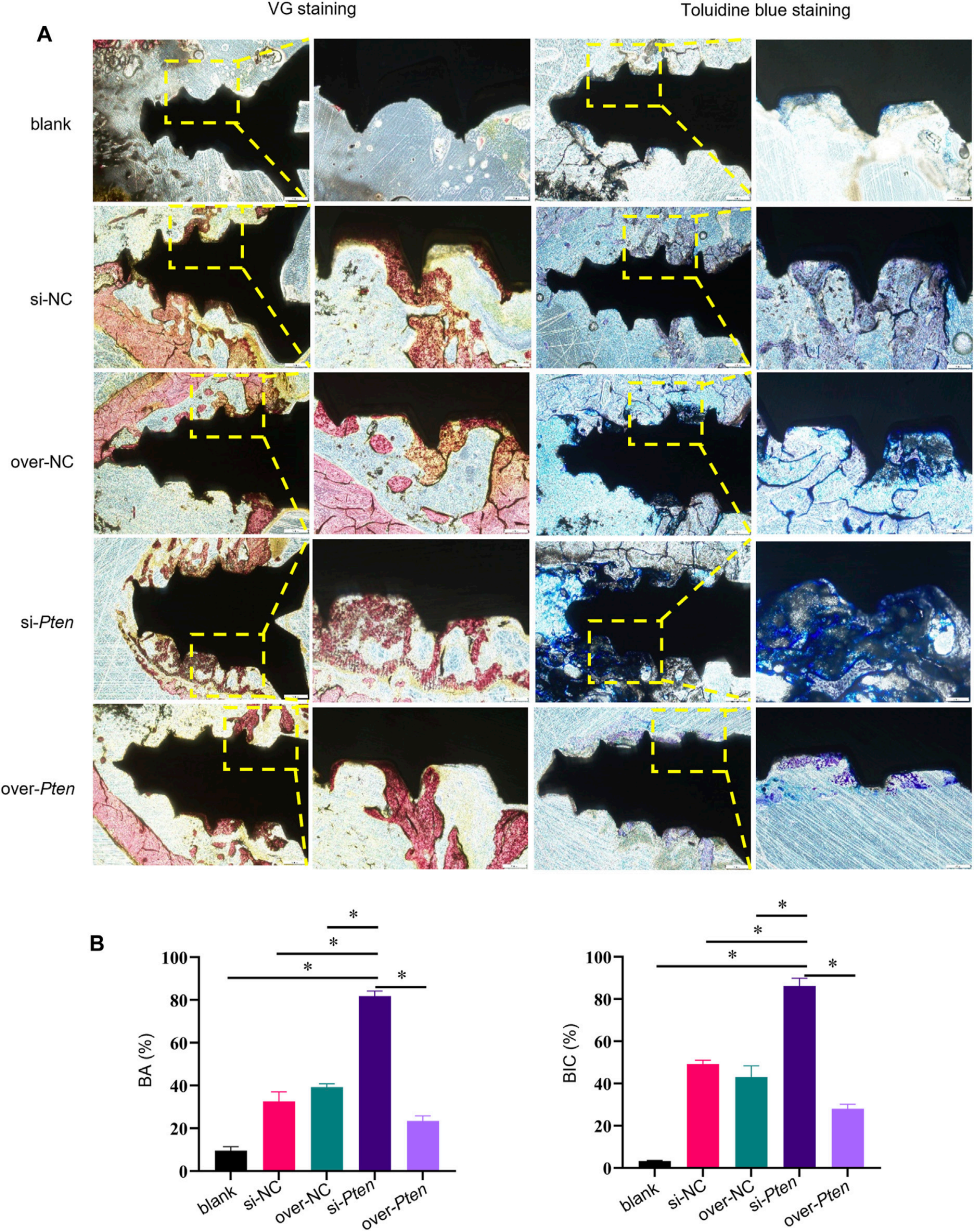
Histological analyses of osseointegration and new bone formation around implants. **(A)** VG and toluidine blue staining, **(B)** Quantification of BA and BIC. Scale bar = 100 μm *p < 0.05.

Staining was performed to evaluate osseointegration and new bone formation to confirm the osteogenesis-promoting effect of TADSCs treated with *Pten* siRNA around the implants. Non-decalcified sections were stained with VG and toluidine blue. In the si-*Pten* group, several bone trabecular structures were in contact with the implant surface. The amount of bone around the implants in the blank group was significantly lower compared to the other groups. Among them, the si-*Pten* group showed the greatest bone volume around the implant, with significantly better surrounding bone quality and higher trabeculae, which were thicker and denser. The amount of bone around the over-*Pten* group was significantly lower compared to the si-*Pten* group and NC groups but higher than the blank group (Figure 6A). The BIC in the blank group was significantly lower than the other groups (*p* < 0.05) but improved significantly in the si-*Pten* group significantly (*p* < 0.05). Quantification of BA around the implant was similar to that of BIC, indicating that TADSC sheets in the si-*Pten* group remarkably enhanced osseointegration around the implant in rats with T2DM (Figure 6B).

### 3.7 Analysis of H&E, masson-trichrome staining, and safranin O-fast green staining

**FIGURE 7:**
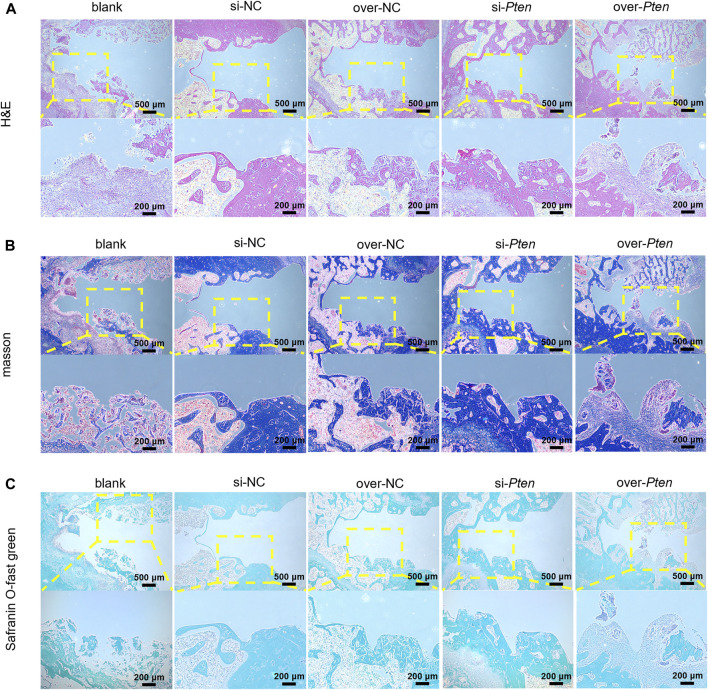
Analysis of H&E, Masson-trichrome, and Safranin O-fast green staining. **(A)** H&E staining results, **(B)** Masson-trichrome staining and **(C)** Safranin O-fast green staining. Scale bar = 100 μm *p < 0.05.

Four weeks after implantation surgery, the peripheral areas of the titanium implant were analyzed by histological examination. The si-*Pten* group revealed significant organized, mineralized bone tissue with a lamellar shape. In contrast, the over-*Pten* group demonstrated a substantial amount of randomly structured, woven bone tissue with large fibrous connective tissue dispersed among the implantation sites (Figure 7A). Next, tissues were subjected to Masson-trichrome staining and Safranin O-fast green staining to evaluate new bone formation and similar phenomena were observed. Through Masson’s trichrome staining, most collagen fiber deposition was observed in the si-*Pten* group, similar to the results of Safranin O-fast green staining, wherein the si-*Pten* group presented an enhanced osseointegration with higher bone formation (staining in green). In contrast, the over-*Pten* group demonstrated lesser new bone formation than the si-*Pten* group but higher compared to the blank group (Figures 7B, C).

### 3.8 TADSC sheets of *Pten* low expression increased M2 phenotype macrophage polarization and attenuated tissue inflammation in T2DM rats

**FIGURE 8:**
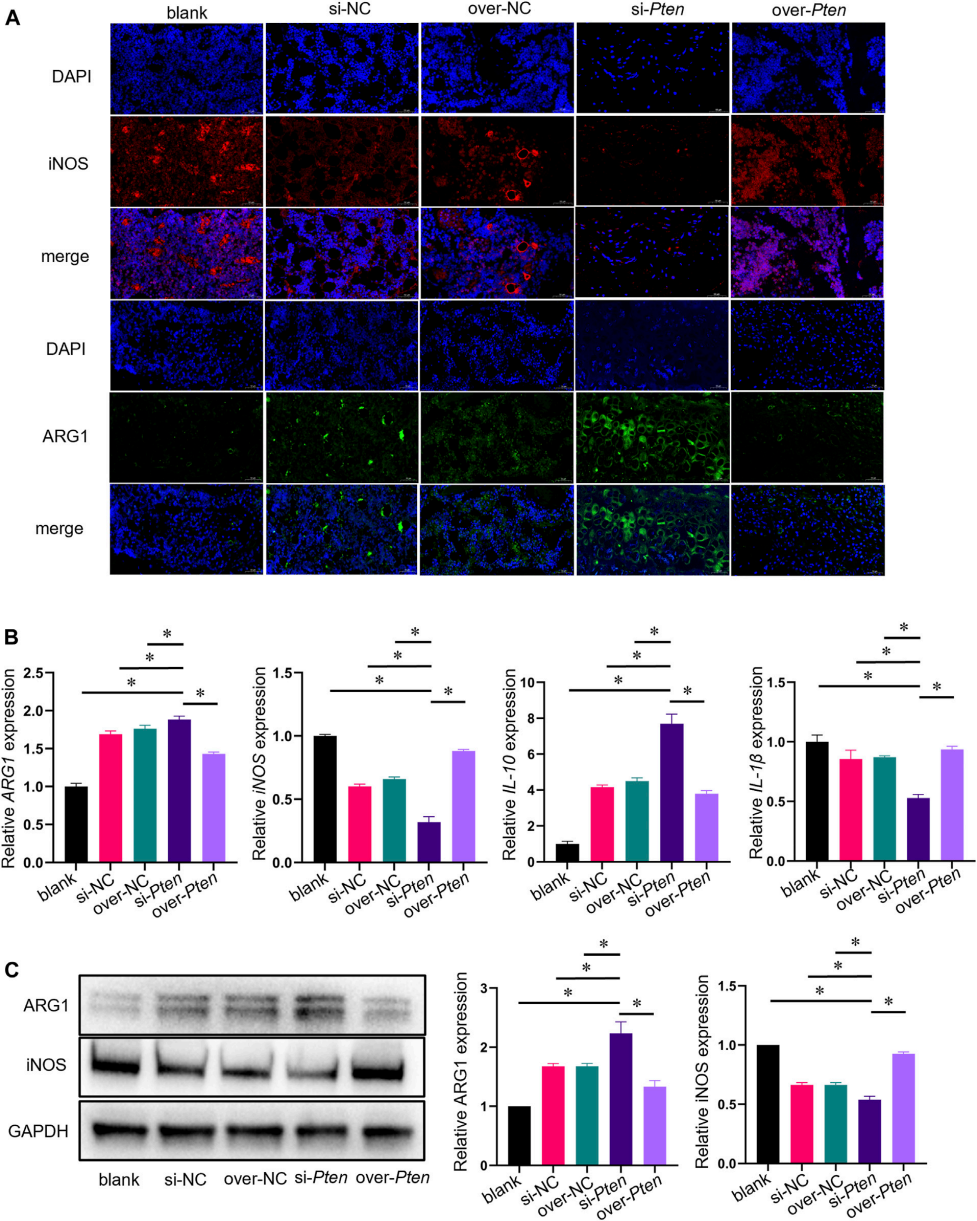
TADSC sheets with low *Pten* expression increase macrophage polarization toward the M2 phenotype and attenuate tissue inflammation in rats with T2DM. **(A)** Representative images of macrophages in tissue sections using immunofluorescent staining (ARG1 in green, iNOS in red, and nuclei in blue; scale bar = 50 μm). **(B)** mRNA expression of marker genes of M1 and M2 macrophages (*Arg1* and *iNOS*), and inflammation-related genes (*IL-10* and *IL-1β*) in the bone tissue around implants. **(C)** Expression of marker proteins of M1 and M2 macrophages in the bone tissue around implants. *p < 0.05.

Next, we investigated whether TADSC sheets treated with *Pten* siRNA could promote M2 macrophage polarization and attenuate inflammation for osteointegration around implants *in vivo*. We analyzed the bone tissues around implants in rats with T2DM. Green-stained cells represent ARG1-positive cells, presenting the M2 macrophages. The proportion of M2 infiltration in the si-*Pten* group was significantly higher compared to other groups. On the contrary, iNOS-positive cells, which represent M1 macrophages, stained red in the si-*Pten* group, were markedly decreased (Figure 8A). qRT-PCR analysis of tissues confirmed that the expression of genes related to M2 phenotype macrophages or anti-inflammation (*Arg1* and *IL-10*) were upregulated, whereas those related to M1 macrophages or inflammation (*iNOS* and *IL-1β*) were downregulated in the si-*Pten* group (Figure 8B). In the si-*Pten* group, the protein expression of ARG1 in the bone tissue around implants was highly expressed, while that of iNOS was suppressed (Figure 8C). *Pten* inhibition in TADSCs could attenuate tissue inflammation around implants in rats with T2DM.

## 4 Discussion

In this study, we showed that the osteogenic capacity of TADSCs derived from rats with T2DM was impaired compared to ADSCs derived from normal rats. PTEN upregulation is important for the attenuated osteogenic capacity in TADSCs owing to the inhibition of the AKT/mTOR/HIF-1α signaling pathway. TADSC sheets treated with *Pten* siRNA reduced tissue inflammation by promoting macrophage polarization toward the M2 phenotype and enhanced osseointegration of titanium implants in rats with T2DM. The upregulation of PTEN in TADSCs was at least partially due to the inhibition of miR-140-3p. Our observations suggest a potential novel clinical strategy of using TADSCs modified by targeting PTEN for promoting implant osseointegration therapy.

The reason for the reduced osteogenic capacity of TADSCs was the downregulation of proteins involved in osteogenesis, such as RUNX2, ALP, BMP2, and COL-1. The AKT/mTOR/HIF-1α signaling pathway, involved in regulating the expression of osteogenic-related proteins, was suppressed. This can be attributed to increased expression of PTEN in TADSCs. As a negative regulator of AKT, PTEN ultimately inhibits the AKT signaling pathway by dephosphorylating AKT, consequently inactivating AKT. This finding was consistent with that of a previous study in which activation of the AKT/mTOR/HIF-1α signaling pathway induced by PTEN inhibition promoted bone regeneration (Yang et al., 2019). We knocked down or overexpressed *Pten* in TADSCs to verify whether PTEN was important for the osteogenic differentiation of TADSCs. Unsurprisingly, the AKT/mTOR/HIF-1α signaling pathway was activated, and the osteogenic ability of TADSCs was enhanced after knocking down *Pten* in TADSCs, and conversely, the osteogenic ability of TADSCs was diminished.

The mechanism underlying PTEN inhibition in TADSCs warrants further investigation. The expression of miRNA-140-3p was suppressed in TADSCs, consistent with a previous study, wherein miR-140-3p levels were reduced in exosomes secreted by diabetic bone marrow mesenchymal stem cells (BMSCs), an important reason for the reduced osteogenic ability of diabetic BMSC-derived exosomes (Wang N. et al., 2022). miR-140-3p can directly target *Pten* and exert a negative regulatory effect in rats (Yang et al., 2021). Thus, we hypothesized that inhibition of miR-140-3p may contribute to the upregulation of PTEN in TADSCs. We treated TADSCs with miR-140-3p mimics and inhibitors to demonstrate this hypothesis. As expected, the upregulation of miR-140-3p induced the suppression of PTEN, while its inhibition upregulated PTEN levels. We inferred that the mechanism underlying PTEN upregulation in TADSCs was at least partially due to the suppression of miRNA-140-3p.

The role of PTEN in regulating the osteogenic capacity of TADSCs improved *in vivo*. TADSC sheets with PTEN overexpression or inhibition were fabricated. Cell sheets were wrapped around the implants and implanted into the femur of rats with T2DM. The results showed the most remarkable enhancement of bone integration around the implant in the siPTEN group, reflected by the parameters used to evaluate bone formation using micro-CT scanning. The peri-implant bone mass, thickness of new bone, and velocity of bone formation were significantly increased after PTEN inhibition in TADSCs. The effect of enhanced bone integration was confirmed by dual fluorescence labeling, H&E staining, Masson’s trichrome staining, Safranin O-fast green staining, and VG and toluidine blue staining. We concluded that PTEN inhibition in TADSC sheets promoted bone integration of peri-implants in rats with T2DM. Although the osteogenic differentiation of TADSCs transfected with *Pten* siRNA was significantly enhanced, an important factor in promoting osseointegration of T2DM implants, accumulating evidence suggests that paracrine cytokines, exosomes, and other active substances are crucial in exerting biological effects of ADSCs (Cai et al., 2020). Osteoblasts and BMSCs, the progenitor cells of osteoblasts, are vital in bone formation (Ning et al., 2022). Thus, we hypothesize exosomes or cytokines secreted by TADSCs modified by knockdown of PTEN can promote the osteogenic differentiation of osteogenic-related cells such as osteoblasts or BMSCs in peri-implant alveolar bone tissue, in turn synergistically promoting implant osseointegration. However, the paracrine factor playing a key role in this process merits further investigation.

The osteogenic capacity of TADSC sheets modified by *Pten* siRNA was significantly enhanced and could directly improve peri-implant osseointegration. Interestingly, overexpression of *Pten* weakened the osteogenic effect of TADSC sheets but was still better compared to the blank group. This suggests that other factors might also be involved in TADSC sheets-induced bone regeneration. ADSCs exerted an immunomodulatory effect that could promote macrophage polarization toward the M2 phenotype through paracrine secretion, in turn inhibiting inflammation and promoting bone regeneration (Shang et al., 2015; Li et al., 2022). Considering the chronic inflammation microenvironment of T2DM (Rohm et al., 2022), we hypothesized that TADSCs could inhibit peri-implant inflammation, consequently promoting peri-implant osseointegration synergistically, and this effect may be enhanced by *Pten* inhibition. To test this hypothesis, we co-cultured TADSCs and macrophages *in vitro* and found that TADSCs promoted M2 polarization of macrophages, which was enhanced by inhibition of *Pten*. In contrast, overexpression of *Pten* markedly attenuated the effect of TADSCs on M2 polarization. Subsequently, this phenomenon was verified *in vivo*. TADSC sheets modified by *Pten* siRNA promoted M2 polarization in peri-implant tissues, and tissue inflammation was suppressed, while TADSC slices over-expressing *Pten* exerted a significantly weaker effect on peri-implant M2 polarization and suppression of inflammation. Therefore, we concluded that knocking down *Pten* could inhibit tissue inflammation and enhance peri-implant osseointegration by promoting macrophage polarization toward the M2 phenotype.

However, some limitations of this study need to be addressed. Implantation of the TADSC sheets into the rat femur was fragile owing to their soft texture. Consequently, the level of osseointegration around the implant can vary. Loading stem cells on various biomaterials, such as hydrogels, could simulate the *in vivo* 3D environment, thus maximizing the regenerative function of stem cells (Xie et al., 2022). The possibility to modify the surface of the implants so stem cells can easily adhere warrants further experimentation.

Taken together, the high expression of PTEN was at least partially due to the downregulation of miR-140-3p in TADSCs, which contributed to the attenuated osteogenic capacity of TADSCs by inhibiting the AKT/mTOR/HIF-1α signaling pathway. TADSC sheets treated with the Pten siRNA inhibited inflammation by promoting macrophage polarization toward the M2 phenotype, which enhanced osteointegration of implants in rats with T2DM. PTEN was crucial in impairing osseointegration, thus offering a novel therapeutic strategy through the modification of stem cells to enhance osseointegration in patients with T2DM.

## 5 Conclusion

In summary, the upregulation of PTEN, partially due to the inhibition of miR-140-3p, is important for the attenuated osteogenic capacity of TADSCs owing to the inhibition of the AKT/mTOR/HIF-1α signaling pathway. Inhibition of PTEN significantly improves the anti-inflammatory effect and osteogenic capacity of TADSCs, further promoting peri-implant bone formation in rats with T2DM. PTEN is critical for inflammatory regulation and osteogenic capacity of TADSCs, suggestive of a potential therapeutic approach for modifying stem cells derived from patients with T2DM to enhance osseointegration.

## Data Availability

The original contributions presented in the study are included in the article/Supplementary Material, further inquiries can be directed to the corresponding authors.

## Abbreviations

ADSCs, adipose-derived stem cells from normal rats; AKT, phosphorylation protein kinase B; ALP, Alkaline phosphatase; BMP2, bone morphogenetic protein 2; COL-1, collagen type I; HIF-1α, hypoxia-inducible factor 1-alpha; p-mTOR, phosphorylated mammalian target of rapamycin; RUNX2, Runt-related transcription factor 2; SD rats, Sprague–Dawley rats; TADSCs, adipose-derived stem cells of T2DM rats; α-MEM, α-minimum essential media.
